# Supplementary material for: Genomic epidemiology of dengue virus 2 and 3 reveals repeated introductions and exportations of several lineages in Colombia
Source: medRxiv. 2025 Nov 3:2025.08.07.25333238. Originally published 2025 Aug 12. Preprint. [Version 3] doi: 10.1101/2025.08.07.25333238 (PMC12363731; doi:10.1101/2025.08.07.25333238)
Supplement: Supplement 1 [file NIHPP2025.08.07.25333238v3-supplement-1.pdf]

medRxiv preprint doi: https://doi.org/10.1101/2025.08.07.25333238; this version posted November 3, 2025. The copyright holder for this preprint (which was not certified by peer review) is the author/funder, who has granted medRxiv a license to display the preprint in perpetuity.
It is made available under a CC-BY 4.0 International license .

Supplemental information index

Figures S1-S4 and their legends in a PDF

Table S1. Accession number and metadata for the sequences generated for this study.

Table S2. GISAID Originating laboratories acknowledgement table.

medRxiv preprint doi: https://doi.org/10.1101/2025.08.07.25333238; this version posted November 3, 2025. The copyright holder for this preprint (which was not certified by peer review) is the author/funder, who has granted medRxiv a license to display the preprint in perpetuity.
It is made available under a CC-BY 4.0 International license .

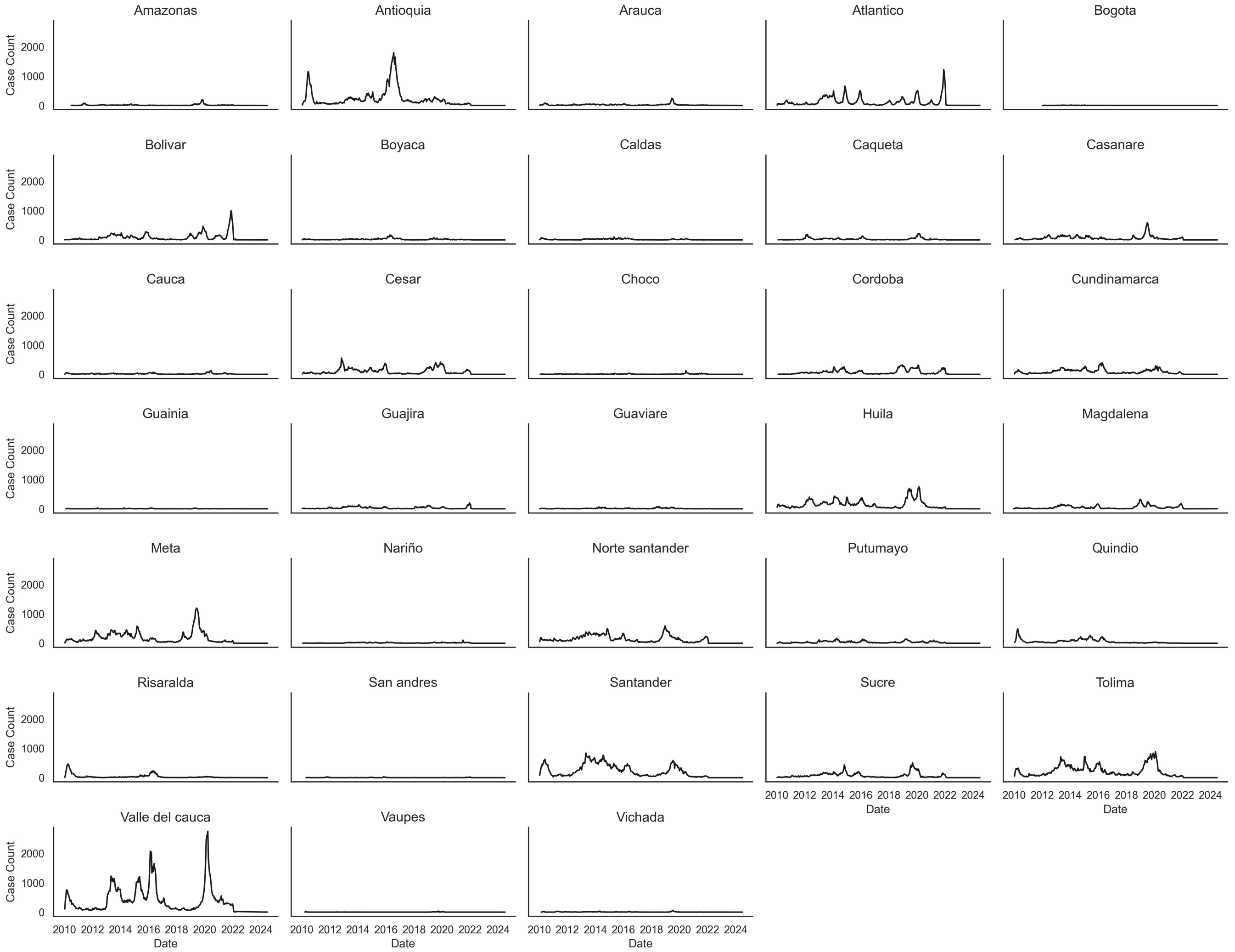

Supplemental figure 1. Number of dengue cases for Colombia's departments.

medRxiv preprint doi: https://doi.org/10.1101/2025.08.07.25333238; this version posted November 3, 2025. The copyright holder for this preprint (which was not certified by peer review) is the author/funder, who has granted medRxiv a license to display the preprint in perpetuity.
It is made available under a CC-BY 4.0 International license .

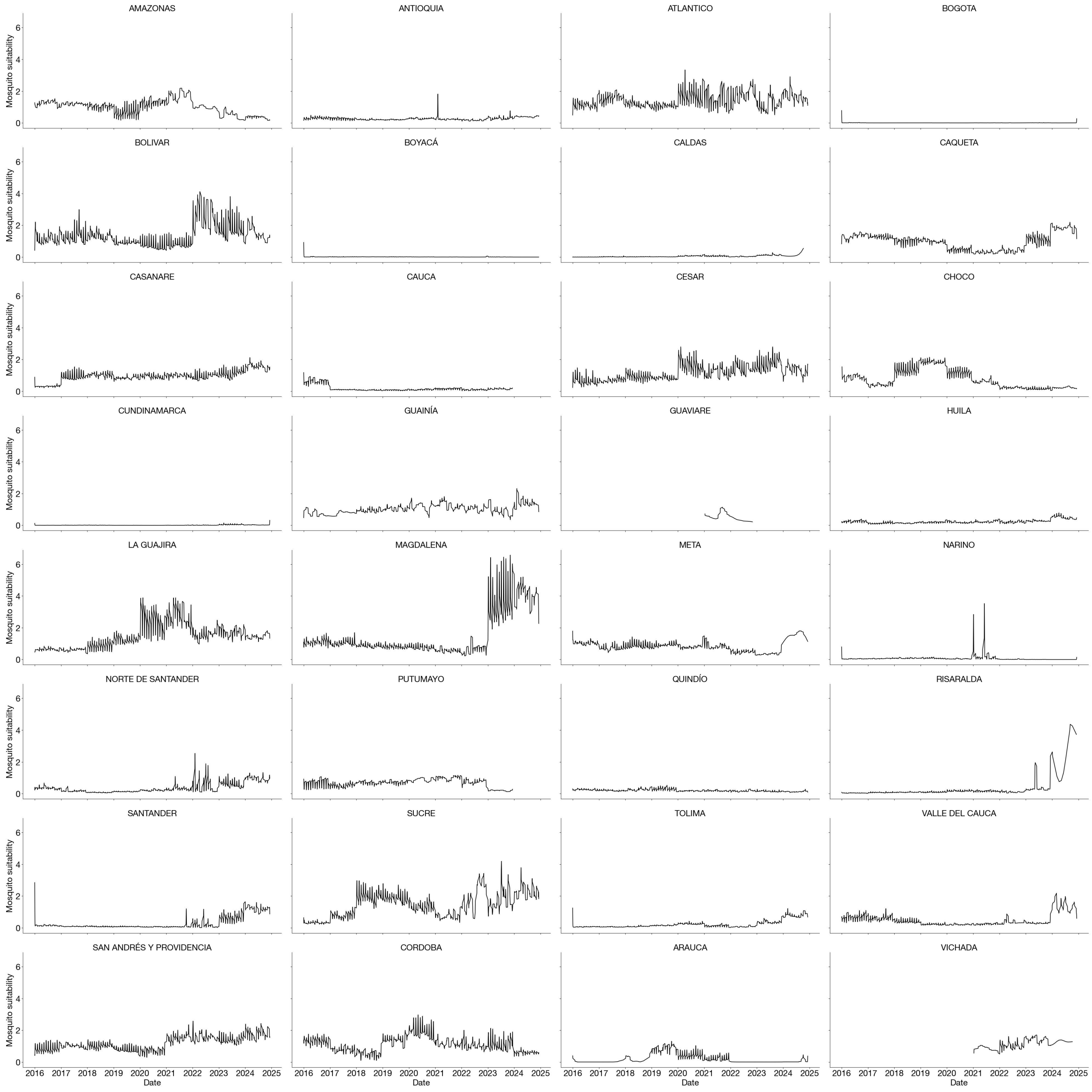

Supplemental figure 2. Mosquito suitability index (index P) for Colombian departments.

medRxiv preprint doi: https://doi.org/10.1101/2025.08.07.25333238; this version posted November 3, 2025. The copyright holder for this preprint (which was not certified by peer review) is the author/funder, who has granted medRxiv a license to display the preprint in perpetuity.
It is made available under a CC-BY 4.0 International license .

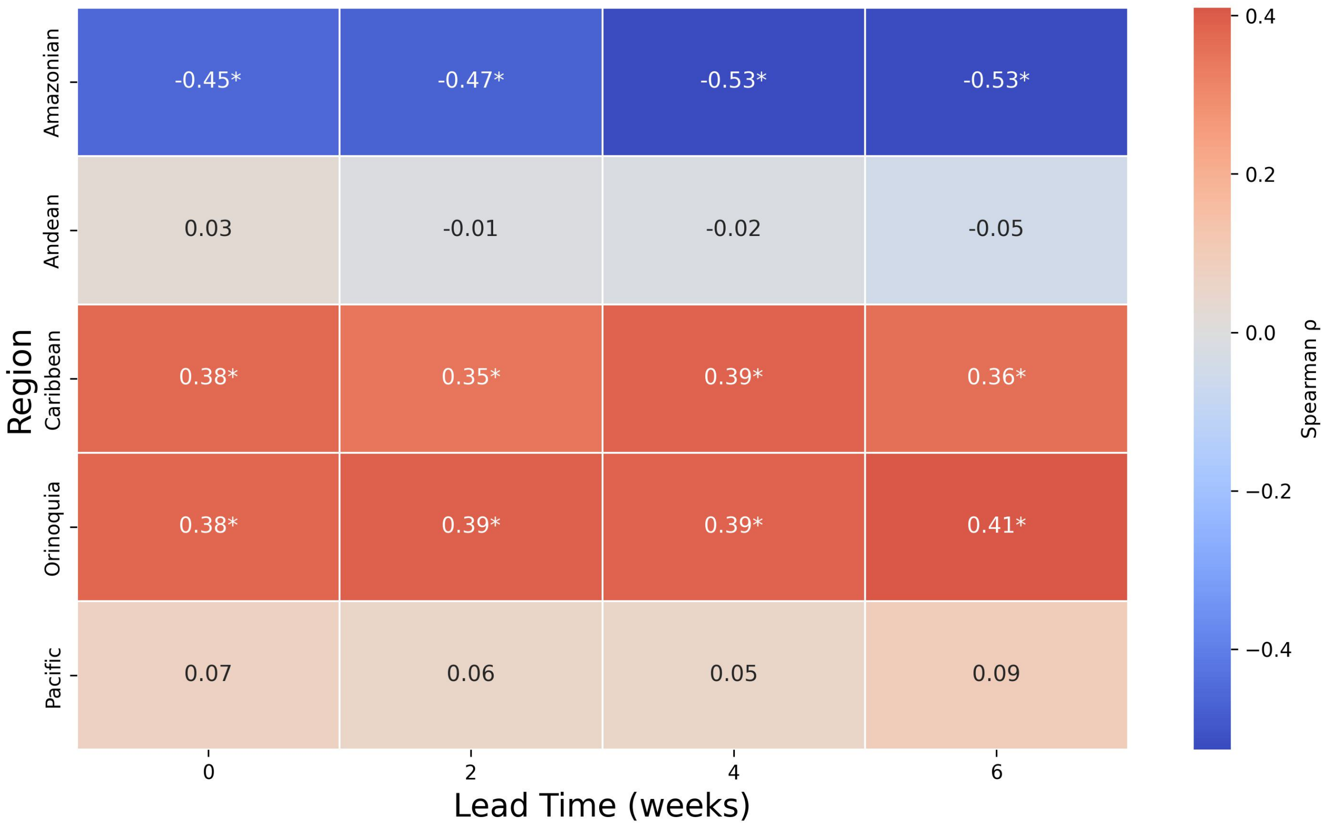

Supplemental figure 3. Correlation plot between index P and dengue cases across the natural regions of Colombia at 0-6 weeks of index P lead time.

The asterisks next to Spearman's ρ values represent significant correlation ($p < 0.05$)

medRxiv preprint doi: https://doi.org/10.1101/2025.08.07.25333238; this version posted November 3, 2025. The copyright holder for this preprint (which was not certified by peer review) is the author/funder, who has granted medRxiv a license to display the preprint in perpetuity.
It is made available under a CC-BY 4.0 International license .

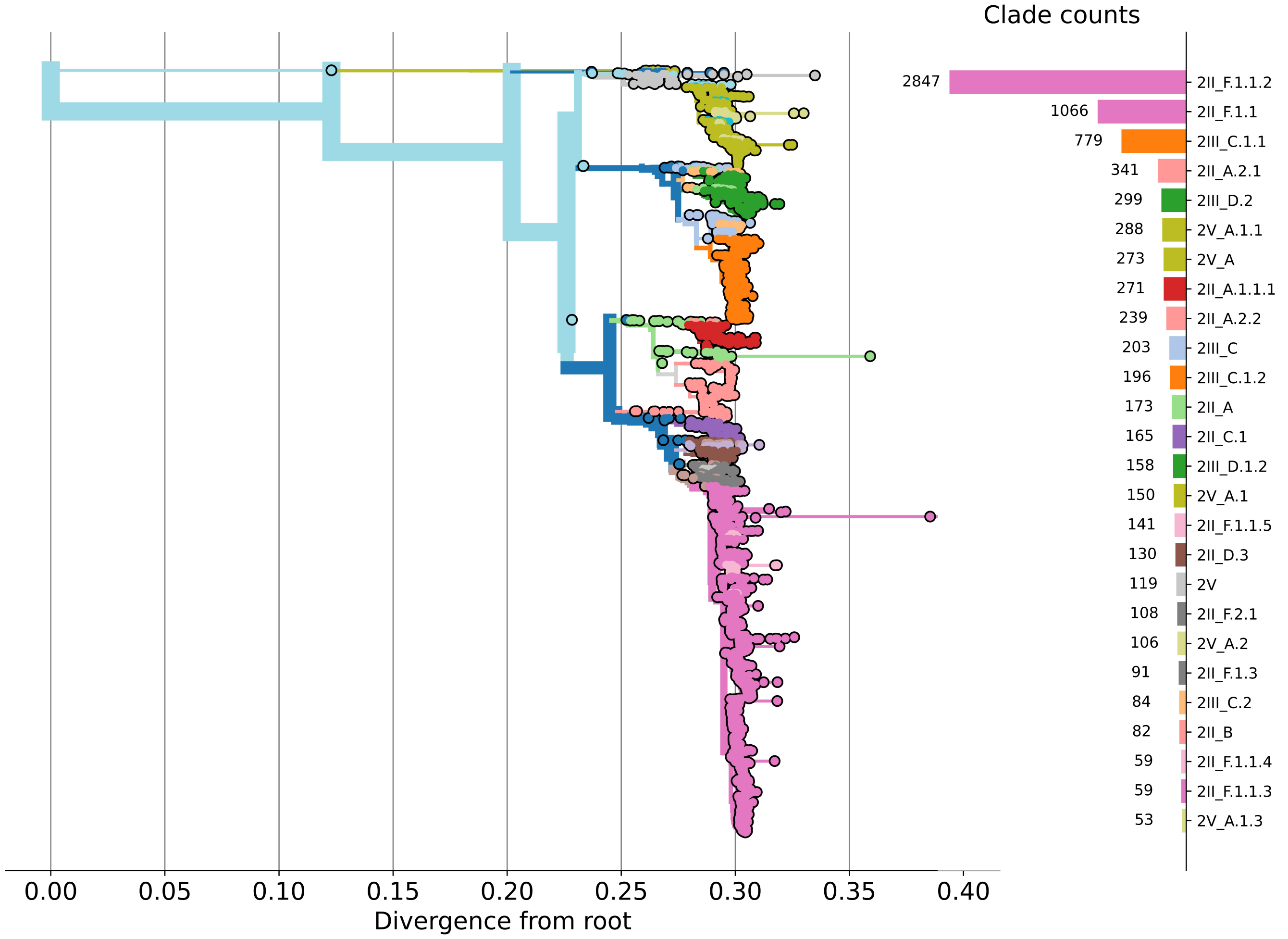

511

Supplemental figure 4. Maximum-likelihood tree of the global diversity of DENV-2 lineages. 512 513

All the DENV-2 genomes publicly available in Epiarbo were downloaded and processed using the Nextclade CLI to classify them according to its lineage, thickness of the branches represent the number of sequences descending from the branch 514 515 516

medRxiv preprint doi: https://doi.org/10.1101/2025.08.07.25333238; this version posted November 3, 2025. The copyright holder for this preprint (which was not certified by peer review) is the author/funder, who has granted medRxiv a license to display the preprint in perpetuity.
It is made available under a CC-BY 4.0 International license .

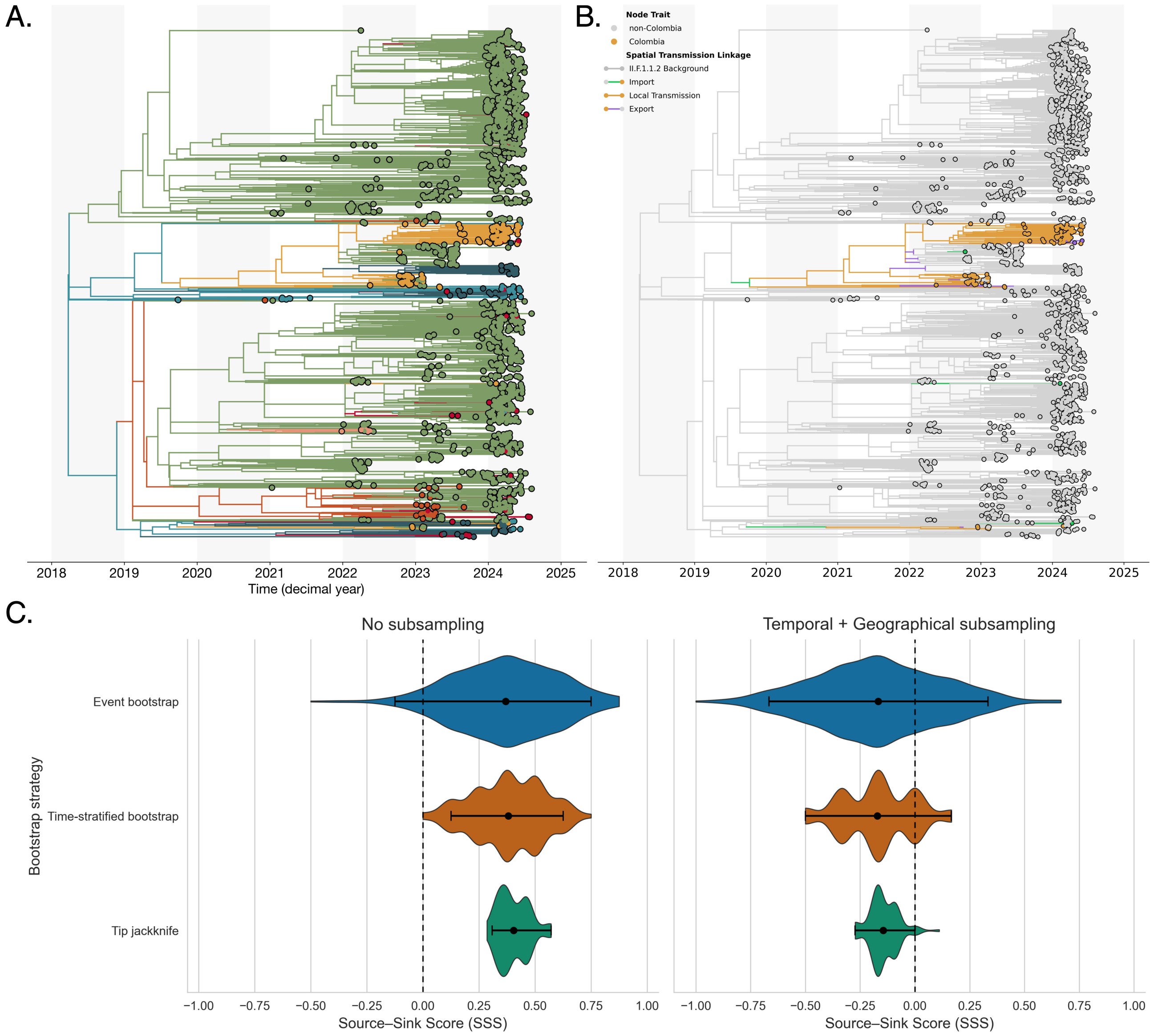

Supplemental figure 5. Phylogeographic structure and robustness of 2_IIF.1.1.2 lineage dissemination in the Americas.

All 1,948 DENV2_II-F.1.1.2 genomes publicly available in EpiArbo were processed using the Nextstrain Dengue build under the mugration model to reconstruct the phylogeographic history of this lineage across the Americas. (A) Maximum-likelihood phylogenetic tree showing Colombian sequences in the context of the continental background, colored by country following the scheme in Figure 3 of the main text. (B) The same tree annotated by Markov-jump transitions through Colombia, with branch colors representing the type of spatial transmission event (importation, local transmission, or export). (C) Bootstrap distributions of the source–sink score (SSS) for Colombia under two independent phylogeographic frameworks: a full maximum-likelihood reconstruction (no subsampling) and a temporally + geographically subsampled BEAST reconstruction. Violin widths represent the density of 5,000 bootstrap replicates for event, time-stratified, and tip-jackknife schemes; dots and horizontal bars denote the mean and 95% CI, and the dashed line marks neutrality ($SSS = 0$). Both frameworks yield small-magnitude, near-neutral SSS values ($|SSS| < 0.4$) with overlapping confidence intervals, indicating that Colombia acts primarily as a hub mediating bidirectional viral exchange rather than as a unidirectional source

medRxiv preprint doi: https://doi.org/10.1101/2025.08.07.25333238; this version posted November 3, 2025. The copyright holder for this preprint (which was not certified by peer review) is the author/funder, who has granted medRxiv a license to display the preprint in perpetuity.
It is made available under a CC-BY 4.0 International license .

or sink.

medRxiv preprint doi: https://doi.org/10.1101/2025.08.07.25333238; this version posted November 3, 2025. The copyright holder for this preprint (which was not certified by peer review) is the author/funder, who has granted medRxiv a license to display the preprint in perpetuity.
It is made available under a CC-BY 4.0 International license .

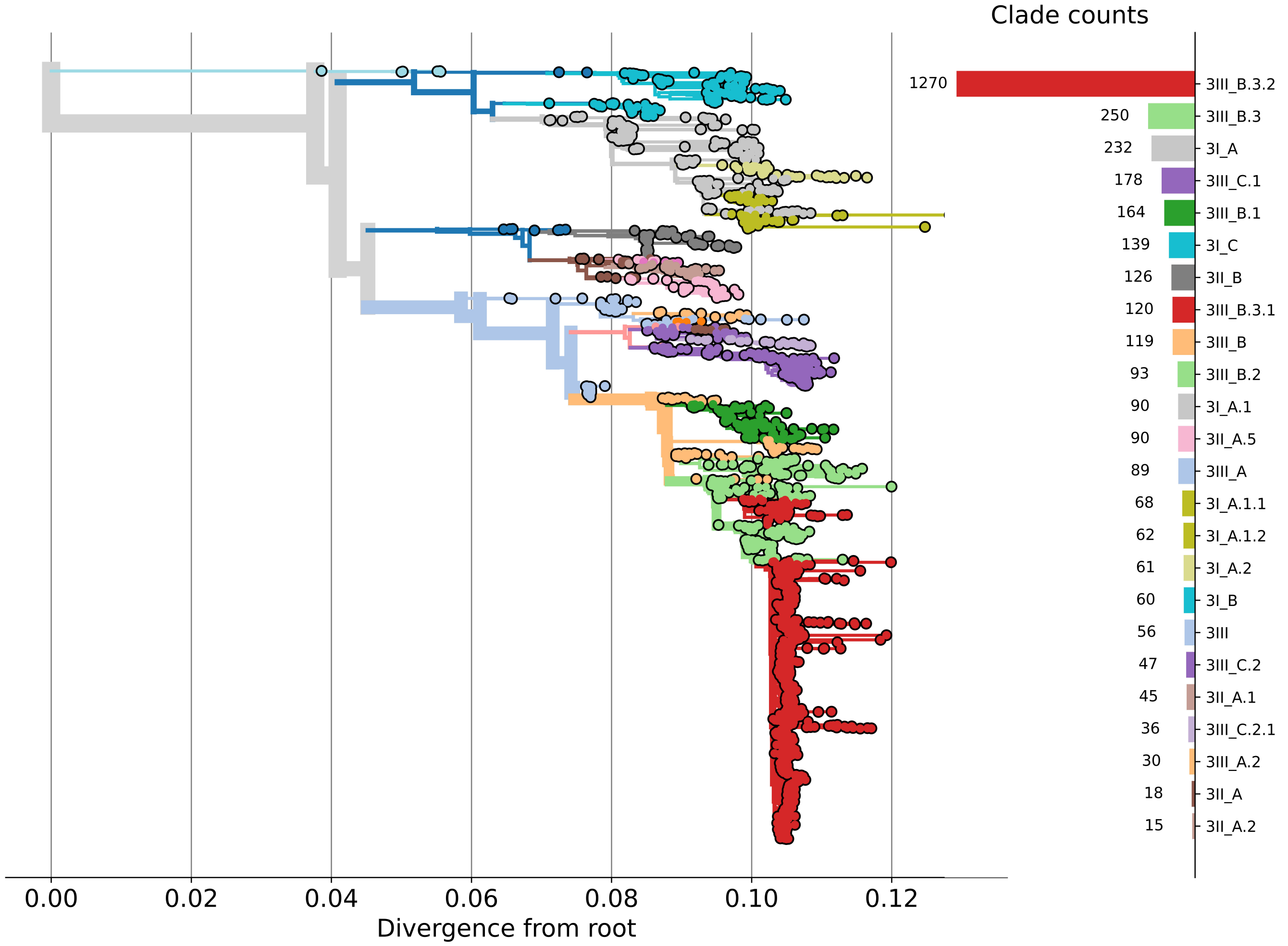

Supplemental figure 6. Maximum-likelihood tree of the global diversity of DENV-3 lineages.

All the DENV-3 genomes publicly available in Epiarbo were downloaded and processed using the Nextclade CLI to classify them according to its lineage, thickness of the branches represent the number of sequences descending from the branch

medRxiv preprint doi: https://doi.org/10.1101/2025.08.07.25333238; this version posted November 3, 2025. The copyright holder for this preprint (which was not certified by peer review) is the author/funder, who has granted medRxiv a license to display the preprint in perpetuity.
It is made available under a CC-BY 4.0 International license .

Study-ID	PCR-CT	Serotype	Lineage	Genome Coverage	Collection Date	City	GenBank-ID
D0348	26.95	DENV2	2II_F.1.1.2	99.07	2024-03-13	Cali	PQ851425
D0350	30.15	DENV2	2II_F.1.1.2	91.33	2024-03-15	Vila Rica, Cauca	PQ851426
D0353	15.24	DENV2	2III_D.2	100.0	2024-03-15	Cali	†
D0360	21.06	DENV2	2III_D.2	100.0	2024-03-20	Candelaria	PQ851427
D0368	25.24	DENV2	2III_D.2	98.0	2024-03-21	Cali	PQ851428
D0370	31.13	DENV2	2III_D.2	91.38	2024-03-26	Cali	PQ851429
D0372	19.96	DENV2	2III_D.2	100.0	2024-03-25	Cali	PQ851430
D0373	18.02	DENV2	2III_D.2	100.0	2024-03-26	Corinto	PQ851431
D0380	26.0	DENV2	2II_F.1.1.2	100.0	2024-04-02	Santander De Quilichao	PQ851432
D0381	11.47	DENV2	2II_F.1.1.2	100.0	2024-04-01	Pradera	PQ851433
D0384	35.01	DENV2	2II_F.1.1.2	68.54	2024-04-02	Cali	PQ851434
D0385	34.19	DENV2	2III_D.2	85.13	2024-04-03	Cali	PQ851435
D0389	28.46	DENV2	2II_F.1.1.2	92.47	2024-04-08	Yumbo	PQ851436
D0394	27.4	DENV2	2II_F.1.1.2	99.6	2024-04-06	Cali	PQ851437
D0397	28.27	DENV2	2III_D.2	94.35	2024-04-08	Cali	PQ851438
D0399	27.44	DENV2	2II_F.1.1.5	99.35	2024-04-10	Cali	PQ851439
D0401	20.82	DENV2	2III_D.2	100.0	2024-04-11	Cali	PQ851440
D0403	34.13	DENV2	2II_F.1.1.2	77.01	2024-04-13	Palmira	PQ851441
D0404	16.75	DENV2	2II_F.1.1.2	100.0	2024-04-11	Cali	PQ851442
D0409	24.26	DENV2	2II_F.1.1.2	100.0	2024-04-18	Cali	PQ851443
D0413	35.22	DENV2	2II_F.1.1.2	64.85	2024-04-17	Cali	*
D0414	31.4	DENV2	2II_F.1.1.2	91.38	2024-04-21	Cali	PQ851444
D0415	29.04	DENV2	2III_D.2	98.95	2024-04-19	Cali	PQ851445
D0417	20.24	DENV2	2II_F.1.1.2	100.0	2024-04-21	Cali	PQ851446
D0421	35.87	DENV2	2II_F.1.1.2	63.97	2024-04-22	Cali	*
D0425	17.16	DENV2	2III_D.2	100.0	2024-04-24	Cali	PQ851447
D0427	33.69	DENV2	2II_F.1.1.2	82.32	2024-04-25	Mariano Ramos	PQ851448
D0430	34.62	DENV2	2II_F.1.1.2	85.09	2024-04-27	Rozo,Valle	PQ851449
D0434	34.61	DENV2	2II_F.1.1.2	57.33	2024-05-05	Bella Vista	*
D0435	30.69	DENV2	2II_F.1.1.2	80.45	2024-05-07	Cali	PQ851450
D0439	28.1	DENV2	2II_F.1.1.2	96.41	2024-05-08	Cali	PQ851451
D0440	28.06	DENV2	2II_F.1.1.2	98.04	2024-05-08	Cali	PQ851452
D0443	27.88	DENV2	2II_F.1.1.2	97.84	2024-05-07	Cali	PQ851453
D0446	22.29	DENV2	2II_F.1.1.2	99.98	2024-05-12	Cali	PQ851454
D0451	34.97	DENV2	2II_F.1.1.2	63.35	2024-05-13	Cali	*
D0456	29.17	DENV2	2III_D.2	97.35	2024-05-15	Corinto	†
D0461	29.18	DENV2	2III_D.2	90.98	2024-05-18	Santander De Quilichao	PQ851455
D0463	29.23	DENV2	2II_F.1.1.2	94.62	2024-05-18	Cali	PQ851456
D0464	15.39	DENV2	2II_F.1.1.2	100.0	2024-05-19	Cali	PQ851457
D0465	23.38	DENV2	2II_F.1.1.2	99.98	2024-05-18	Cali	PQ851458
D0467	32.99	DENV2	2II_F.1.1.2	87.53	2024-05-23	Yotoco	PQ851459
D0471	23.64	DENV2	2II_F.1.1.2	99.98	2024-05-25	Cali	PQ851460
D0473	26.89	DENV2	2II_F.1.1.2	97.05	2024-05-26	Cali	PQ851461
D0480	31.59	DENV2	2III_D.2	84.52	2024-05-30	Cali	PQ851462
D0489	28.56	DENV2	2II_F.1.1.2	92.94	2024-06-06	Cali	PQ851463
D0491	26.97	DENV2	2II_F.1.1.2	99.59	2024-06-07	Palmira	PQ851464
D0495	27.36	DENV2	2II_F.1.1.2	98.6	2024-06-11	Cali	PQ851465
D0498	32.55	DENV2	2III_D.2	76.06	2024-06-10	Cali	PQ851466
D0499	32.83	DENV2	2II_F.1.1.2	71.8	2024-06-09	Vijes	PQ851467
D0523	21.94	DENV2	2II_F.1.1.2	97.05	2024-06-20	Palmira	PQ851468
D0524	21.62	DENV2	2II_F.1.1.2	98.52	2024-06-22	Cali	PQ851469
D0525	20.46	DENV2	2II_F.1.1.2	99.58	2024-06-23	Cali	PQ851470
D0526	17.27	DENV2	2II_F.1.1.2	99.78	2024-06-23	Cali	PQ851471
D0533	19.34	DENV2	2II_F.1.1.2	100.0	2024-06-27	Jamundi	PQ851472
D0537	31.09	DENV2	2II_F.1.1.2	81.02	2024-07-02	Cali	PQ851473
D0540	25.03	DENV2	2II_F.1.1.2	95.6	2024-07-05	Candelaria	PQ851474
D0544	28.31	DENV2	2II_F.1.1.2	87.77	2024-07-13	Cali	PQ851475
D0550	20.83	DENV2	2II_F.1.1.2	99.78	2024-07-20	Cali	PQ851476
D0560	30.38	DENV2	2II_F.1.1.2	87.69	2024-07-26	Cali	PQ851477
D0566	17.59	DENV2	2II_F.1.1.2	100.0	2024-07-31	Cali	PQ851478
D0567	17.88	DENV2	2II_F.1.1.2	100.0	2024-07-28	Cali	PQ851479
D0568	32.74	DENV2	2II_F.1.1.2	72.44	2024-08-01	Cali	PQ851480
D0569	26.87	DENV2	2II_F.1.1.2	93.45	2024-08-01	Cali	PQ851481
D0573	31.1	DENV2	2II_F.1.1.2	82.25	2024-08-03	Caloto, Cauca	PQ851482
D0575		DENV2	2II_F.1.1.2	85.86	2024-08-07	Putumayo	PQ851483
D0576	34.43	DENV2	2II_F.1.1.2	86.45	2024-08-07	Santander De Quilichao	PQ851484
D0577	26.24	DENV2	2II_F.1.1.5	98.61	2024-08-06	Cali	PQ851485
D0578	25.33	DENV2	2II_F.1.1.2	92.89	2024-08-07	Cali	PQ851486
D0476	28.5	DENV2	2II_F.1.1.2	95.93	2024-05-28	Cali	PQ851487
D0478	24.2	DENV2	2II_F.1.1.2	100.0	2024-05-28	Cali	PQ851488
D0585	33.19	DENV2	2II_F.1.1.2	65.44	2024-08-14	Cali	*
D0586	33.3	DENV2	2II_F.1.1.2	86.41	2024-08-15	Cali	PQ851489
D0587	31.47	DENV2	2II_F.1.1.2	89.35	2024-08-08	Cali	PQ851490

medRxiv preprint doi: https://doi.org/10.1101/2025.08.07.25333238; this version posted November 3, 2025. The copyright holder for this preprint (which was not certified by peer review) is the author/funder, who has granted medRxiv a license to display the preprint in perpetuity.
It is made available under a CC-BY 4.0 International license .

Notes:

* %Coverage below the threshold for submission; † Suspected co-infection or cross-contamination
