# Supplementary material for: Genomic epidemiology of dengue virus 2 and 3 reveals repeated introductions and exportations of several lineages in Colombia
Source: medRxiv. 2025 Aug 26:2025.08.07.25333238. Originally published 2025 Aug 12. Preprint. [Version 2] doi: 10.1101/2025.08.07.25333238 (PMC12363731; doi:10.1101/2025.08.07.25333238)
Supplement: Supplementary file 1 [file NIHPP2025.08.07.25333238v2-supplement-1.pdf]

medRxiv preprint doi: https://doi.org/10.1101/2025.08.07.25333238; this version posted August 26, 2025. The copyright holder for this preprint (which was not certified by peer review) is the author/funder, who has granted medRxiv a license to display the preprint in perpetuity.
It is made available under a CC-BY 4.0 International license .

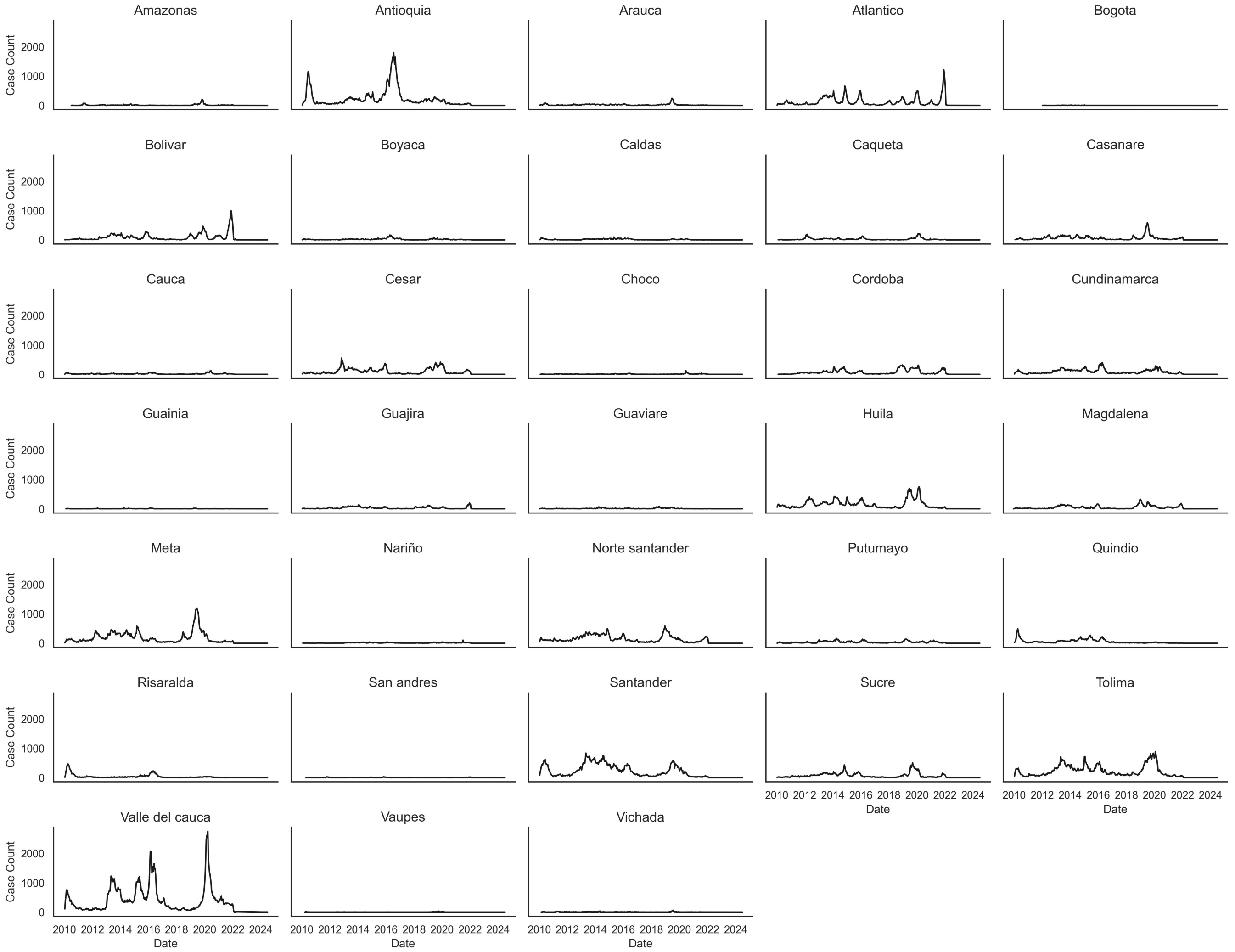


**Supplemental figure 1. Number of dengue cases for Colombia's departments.**

medRxiv preprint doi: https://doi.org/10.1101/2025.08.07.25333238; this version posted August 26, 2025. The copyright holder for this preprint (which was not certified by peer review) is the author/funder, who has granted medRxiv a license to display the preprint in perpetuity.
It is made available under a CC-BY 4.0 International license .

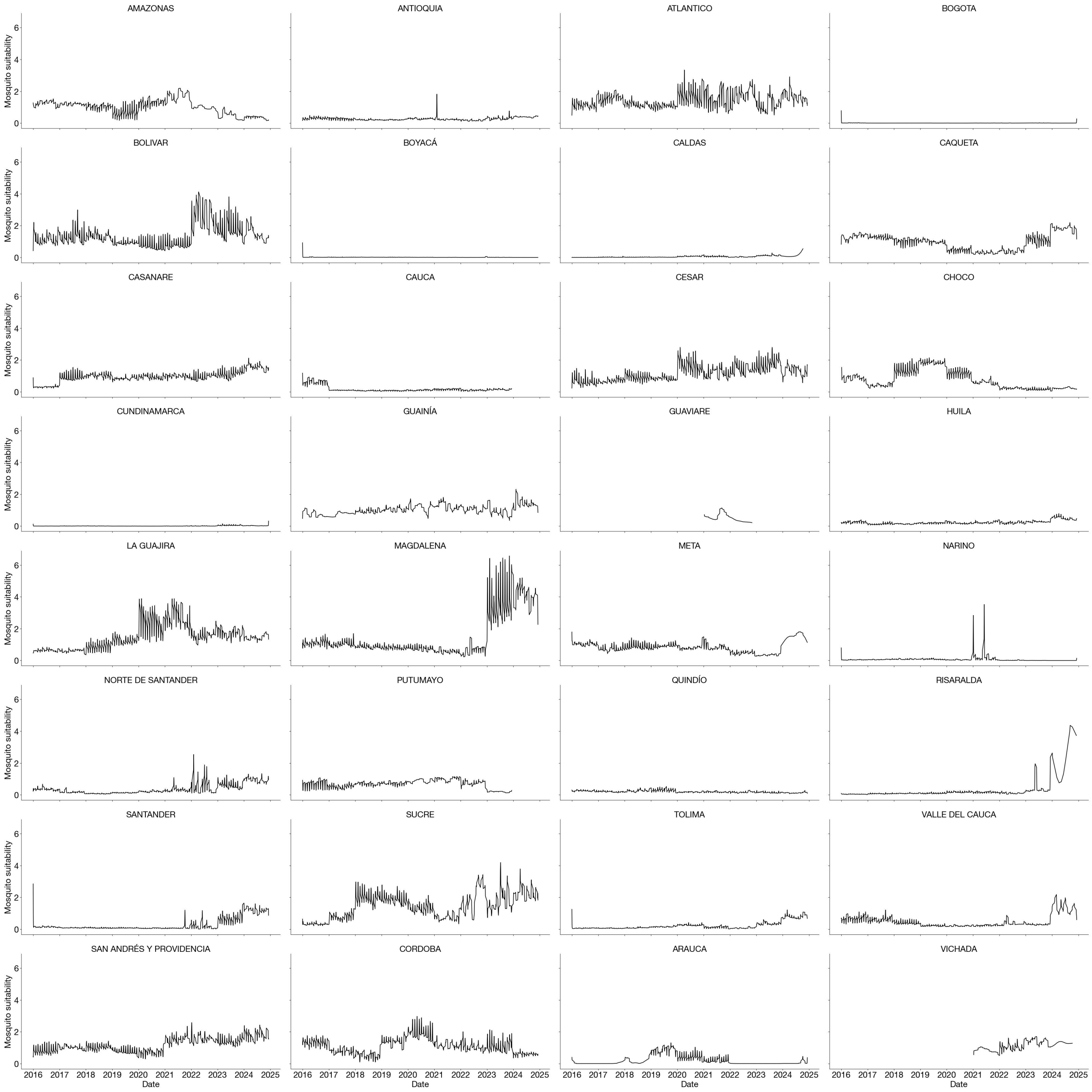


**Supplemental figure 2. Mosquito suitability index (index P) for Colombian departments.**

medRxiv preprint doi: https://doi.org/10.1101/2025.08.07.25333238; this version posted August 26, 2025. The copyright holder for this preprint (which was not certified by peer review) is the author/funder, who has granted medRxiv a license to display the preprint in perpetuity.
It is made available under a CC-BY 4.0 International license .

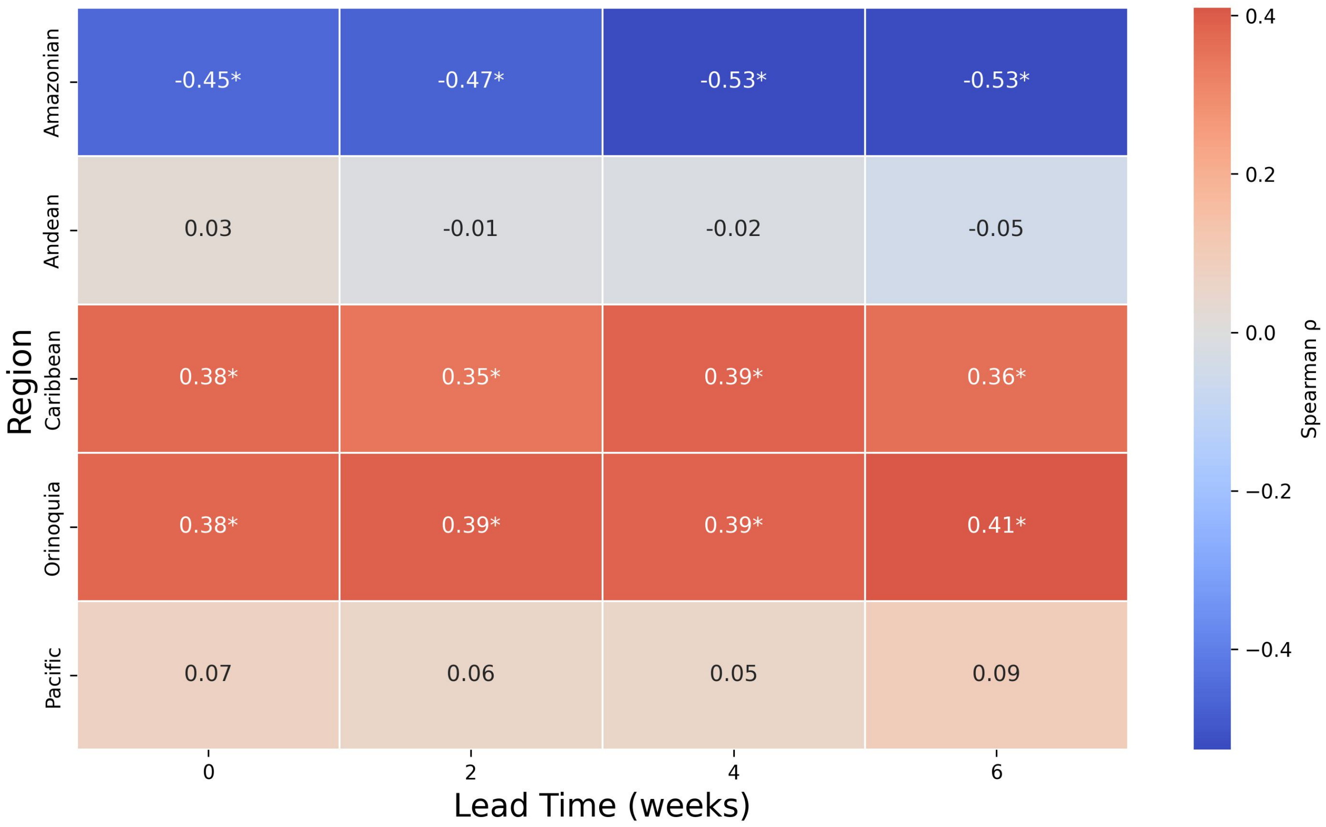


**Supplemental figure 3. Correlation plot between index P and dengue cases across the natural regions of Colombia at 0-6 weeks of index P lead time.**

The asterisks next to Spearman's $\rho$ values represent significant correlation $(p < 0.05)$

medRxiv preprint doi: https://doi.org/10.1101/2025.08.07.25333238; this version posted August 26, 2025. The copyright holder for this preprint (which was not certified by peer review) is the author/funder, who has granted medRxiv a license to display the preprint in perpetuity.
It is made available under a CC-BY 4.0 International license .

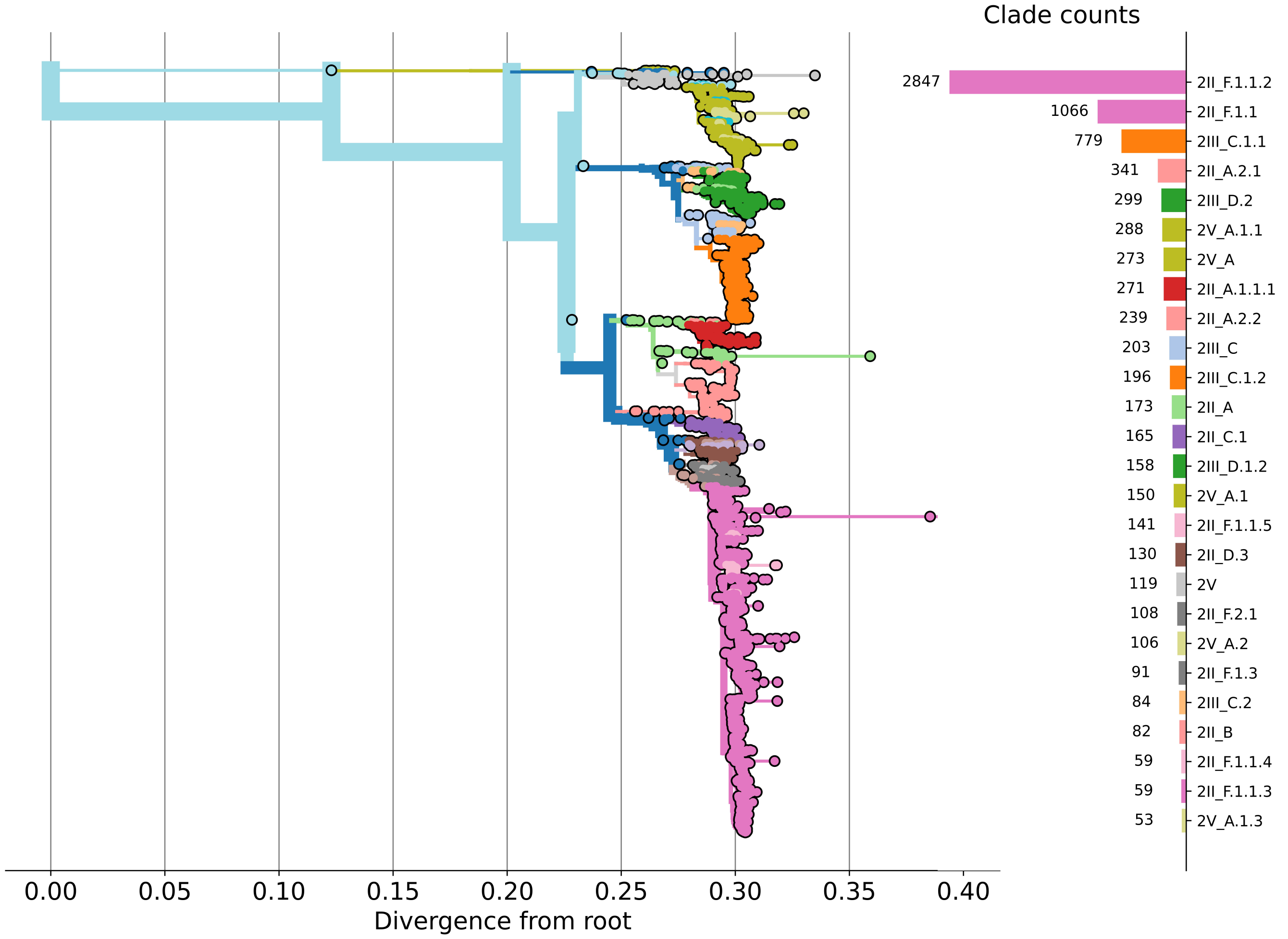


## Supplemental figure 4. Maximum-likelihood tree of the global diversity of DENV-2 lineages.

All the DENV-2 genomes publicly available in Epiarbo were downloaded and processed using the Nextclade CLI to classify them according to its lineage, thickness of the branches represent the number of sequences descending from the branch

medRxiv preprint doi: https://doi.org/10.1101/2025.08.07.25333238; this version posted August 26, 2025. The copyright holder for this preprint (which was not certified by peer review) is the author/funder, who has granted medRxiv a license to display the preprint in perpetuity.
It is made available under a CC-BY 4.0 International license .

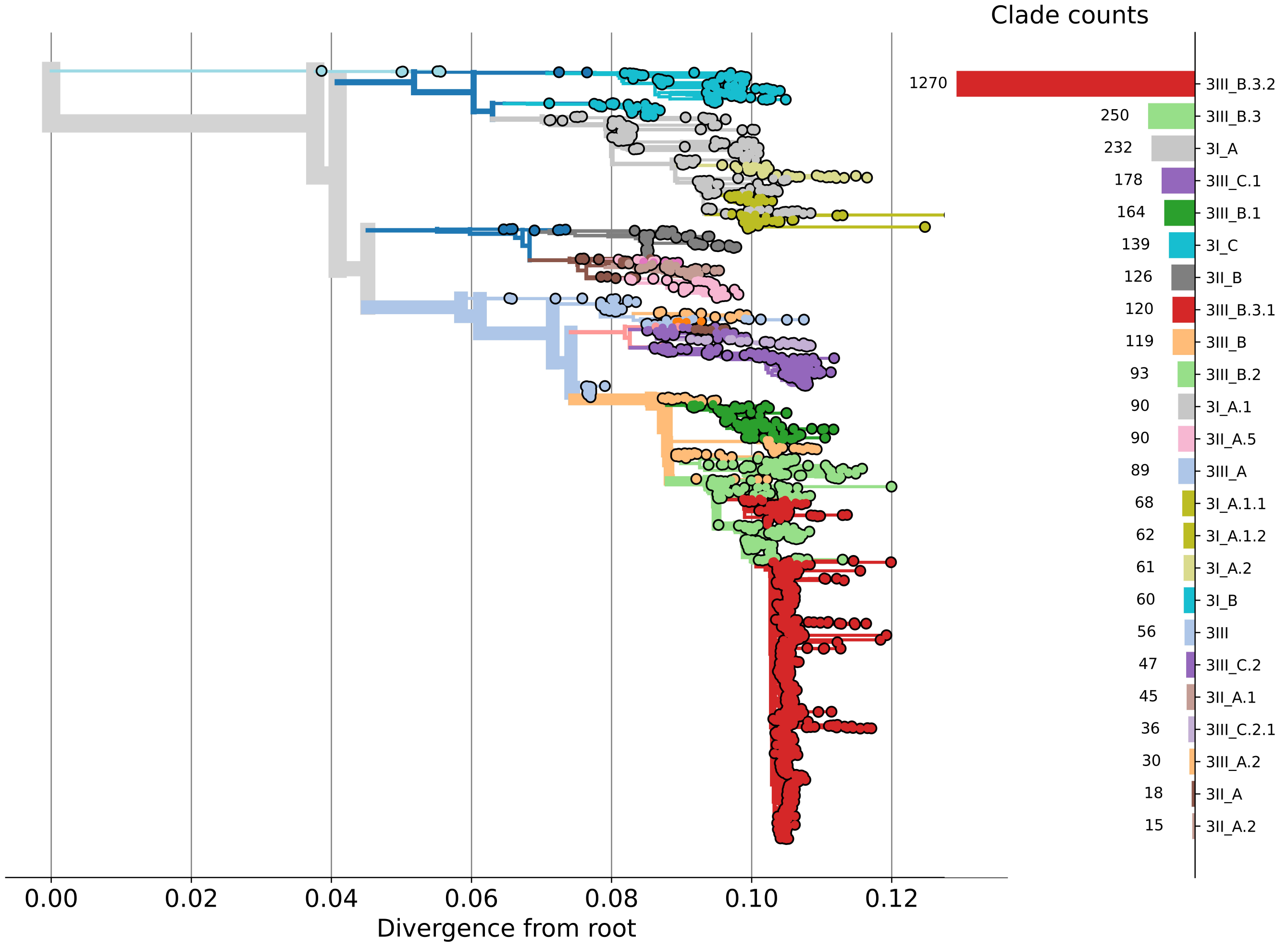


## Supplemental figure 5. Maximum-likelihood tree of the global diversity of DENV-3 lineages.

All the DENV-3 genomes publicly available in Epiarbo were downloaded and processed using the Nextclade CLI to classify them according to its lineage, thickness of the branches represent the number of sequences descending from the branch

medRxiv preprint doi: https://doi.org/10.1101/2025.08.07.25333238; this version posted August 26, 2025. The copyright holder for this preprint (which was not certified by peer review) is the author/funder, who has granted medRxiv a license to display the preprint in perpetuity.
It is made available under a CC-BY 4.0 International license .

| Study-ID | PCR-CT | Serotype | Lineage | Genome Coverage | Collection Date | City | GenBank-ID |
|---|---|---|---|---|---|---|---|
| D0348 | 26.95 | DENV2 | 2II_F.1.1.2 | 99.07 | 2024-03-13 | Cali | PQ851425 |
| D0350 | 30.15 | DENV2 | 2II_F.1.1.2 | 91.33 | 2024-03-15 | Vila Rica, Cauca | PQ851426 |
| D0353 | 15.24 | DENV2 | 2III_D.2 | 100.0 | 2024-03-15 | Cali | † |
| D0360 | 21.06 | DENV2 | 2III_D.2 | 100.0 | 2024-03-20 | Candelaria | PQ851427 |
| D0368 | 25.24 | DENV2 | 2III_D.2 | 98.0 | 2024-03-21 | Cali | PQ851428 |
| D0370 | 31.13 | DENV2 | 2III_D.2 | 91.38 | 2024-03-26 | Cali | PQ851429 |
| D0372 | 19.96 | DENV2 | 2III_D.2 | 100.0 | 2024-03-25 | Cali | PQ851430 |
| D0373 | 18.02 | DENV2 | 2III_D.2 | 100.0 | 2024-03-26 | Corinto | PQ851431 |
| D0380 | 26.0 | DENV2 | 2II_F.1.1.2 | 100.0 | 2024-04-02 | Santander De Quilichao | PQ851432 |
| D0381 | 11.47 | DENV2 | 2II_F.1.1.2 | 100.0 | 2024-04-01 | Pradera | PQ851433 |
| D0384 | 35.01 | DENV2 | 2II_F.1.1.2 | 68.54 | 2024-04-02 | Cali | PQ851434 |
| D0385 | 34.19 | DENV2 | 2III_D.2 | 85.13 | 2024-04-03 | Cali | PQ851435 |
| D0389 | 28.46 | DENV2 | 2II_F.1.1.2 | 92.47 | 2024-04-08 | Yumbo | PQ851436 |
| D0394 | 27.4 | DENV2 | 2II_F.1.1.2 | 99.6 | 2024-04-06 | Cali | PQ851437 |
| D0397 | 28.27 | DENV2 | 2III_D.2 | 94.35 | 2024-04-08 | Cali | PQ851438 |
| D0399 | 27.44 | DENV2 | 2II_F.1.1.5 | 99.35 | 2024-04-10 | Cali | PQ851439 |
| D0401 | 20.82 | DENV2 | 2III_D.2 | 100.0 | 2024-04-11 | Cali | PQ851440 |
| D0403 | 34.13 | DENV2 | 2II_F.1.1.2 | 77.01 | 2024-04-13 | Palmira | PQ851441 |
| D0404 | 16.75 | DENV2 | 2II_F.1.1.2 | 100.0 | 2024-04-11 | Cali | PQ851442 |
| D0409 | 24.26 | DENV2 | 2II_F.1.1.2 | 100.0 | 2024-04-18 | Cali | PQ851443 |
| D0413 | 35.22 | DENV2 | 2II_F.1.1.2 | 64.85 | 2024-04-17 | Cali | * |
| D0414 | 31.4 | DENV2 | 2II_F.1.1.2 | 91.38 | 2024-04-21 | Cali | PQ851444 |
| D0415 | 29.04 | DENV2 | 2III_D.2 | 98.95 | 2024-04-19 | Cali | PQ851445 |
| D0417 | 20.24 | DENV2 | 2II_F.1.1.2 | 100.0 | 2024-04-21 | Cali | PQ851446 |
| D0421 | 35.87 | DENV2 | 2II_F.1.1.2 | 63.97 | 2024-04-22 | Cali | * |
| D0425 | 17.16 | DENV2 | 2III_D.2 | 100.0 | 2024-04-24 | Cali | PQ851447 |
| D0427 | 33.69 | DENV2 | 2II_F.1.1.2 | 82.32 | 2024-04-25 | Mariano Ramos | PQ851448 |
| D0430 | 34.62 | DENV2 | 2II_F.1.1.2 | 85.09 | 2024-04-27 | Rozo,Valle | PQ851449 |
| D0434 | 34.61 | DENV2 | 2II_F.1.1.2 | 57.33 | 2024-05-05 | Bella Vista | * |
| D0435 | 30.69 | DENV2 | 2II_F.1.1.2 | 80.45 | 2024-05-07 | Cali | PQ851450 |
| D0439 | 28.1 | DENV2 | 2II_F.1.1.2 | 96.41 | 2024-05-08 | Cali | PQ851451 |
| D0440 | 28.06 | DENV2 | 2II_F.1.1.2 | 98.04 | 2024-05-08 | Cali | PQ851452 |
| D0443 | 27.88 | DENV2 | 2II_F.1.1.2 | 97.84 | 2024-05-07 | Cali | PQ851453 |
| D0446 | 22.29 | DENV2 | 2II_F.1.1.2 | 99.98 | 2024-05-12 | Cali | PQ851454 |
| D0451 | 34.97 | DENV2 | 2II_F.1.1.2 | 63.35 | 2024-05-13 | Cali | * |
| D0456 | 29.17 | DENV2 | 2III_D.2 | 97.35 | 2024-05-15 | Corinto | † |
| D0461 | 29.18 | DENV2 | 2III_D.2 | 90.98 | 2024-05-18 | Santander De Quilichao | PQ851455 |
| D0463 | 29.23 | DENV2 | 2II_F.1.1.2 | 94.62 | 2024-05-18 | Cali | PQ851456 |
| D0464 | 15.39 | DENV2 | 2II_F.1.1.2 | 100.0 | 2024-05-19 | Cali | PQ851457 |
| D0465 | 23.38 | DENV2 | 2II_F.1.1.2 | 99.98 | 2024-05-18 | Cali | PQ851458 |
| D0467 | 32.99 | DENV2 | 2II_F.1.1.2 | 87.53 | 2024-05-23 | Yotoco | PQ851459 |
| D0471 | 23.64 | DENV2 | 2II_F.1.1.2 | 99.98 | 2024-05-25 | Cali | PQ851460 |
| D0473 | 26.89 | DENV2 | 2II_F.1.1.2 | 97.05 | 2024-05-26 | Cali | PQ851461 |
| D0480 | 31.59 | DENV2 | 2III_D.2 | 84.52 | 2024-05-30 | Cali | PQ851462 |
| D0489 | 28.56 | DENV2 | 2II_F.1.1.2 | 92.94 | 2024-06-06 | Cali | PQ851463 |
| D0491 | 26.97 | DENV2 | 2II_F.1.1.2 | 99.59 | 2024-06-07 | Palmira | PQ851464 |
| D0495 | 27.36 | DENV2 | 2II_F.1.1.2 | 98.6 | 2024-06-11 | Cali | PQ851465 |
| D0498 | 32.55 | DENV2 | 2III_D.2 | 76.06 | 2024-06-10 | Cali | PQ851466 |
| D0499 | 32.83 | DENV2 | 2II_F.1.1.2 | 71.8 | 2024-06-09 | Vijes | PQ851467 |
| D0523 | 21.94 | DENV2 | 2II_F.1.1.2 | 97.05 | 2024-06-20 | Palmira | PQ851468 |
| D0524 | 21.62 | DENV2 | 2II_F.1.1.2 | 98.52 | 2024-06-22 | Cali | PQ851469 |
| D0525 | 20.46 | DENV2 | 2II_F.1.1.2 | 99.58 | 2024-06-23 | Cali | PQ851470 |
| D0526 | 17.27 | DENV2 | 2II_F.1.1.2 | 99.78 | 2024-06-23 | Cali | PQ851471 |
| D0533 | 19.34 | DENV2 | 2II_F.1.1.2 | 100.0 | 2024-06-27 | Jamundi | PQ851472 |
| D0537 | 31.09 | DENV2 | 2II_F.1.1.2 | 81.02 | 2024-07-02 | Cali | PQ851473 |
| D0540 | 25.03 | DENV2 | 2II_F.1.1.2 | 95.6 | 2024-07-05 | Candelaria | PQ851474 |
| D0544 | 28.31 | DENV2 | 2II_F.1.1.2 | 87.77 | 2024-07-13 | Cali | PQ851475 |
| D0550 | 20.83 | DENV2 | 2II_F.1.1.2 | 99.78 | 2024-07-20 | Cali | PQ851476 |
| D0560 | 30.38 | DENV2 | 2II_F.1.1.2 | 87.69 | 2024-07-26 | Cali | PQ851477 |
| D0566 | 17.59 | DENV2 | 2II_F.1.1.2 | 100.0 | 2024-07-31 | Cali | PQ851478 |
| D0567 | 17.88 | DENV2 | 2II_F.1.1.2 | 100.0 | 2024-07-28 | Cali | PQ851479 |
| D0568 | 32.74 | DENV2 | 2II_F.1.1.2 | 72.44 | 2024-08-01 | Cali | PQ851480 |
| D0569 | 26.87 | DENV2 | 2II_F.1.1.2 | 93.45 | 2024-08-01 | Cali | PQ851481 |
| D0573 | 31.1 | DENV2 | 2II_F.1.1.2 | 82.25 | 2024-08-03 | Caloto, Cauca | PQ851482 |
| D0575 | | DENV2 | 2II_F.1.1.2 | 85.86 | 2024-08-07 | Putumayo | PQ851483 |
| D0576 | 34.43 | DENV2 | 2II_F.1.1.2 | 86.45 | 2024-08-07 | Santander De Quilichao | PQ851484 |
| D0577 | 26.24 | DENV2 | 2II_F.1.1.5 | 98.61 | 2024-08-06 | Cali | PQ851485 |
| D0578 | 25.33 | DENV2 | 2II_F.1.1.2 | 92.89 | 2024-08-07 | Cali | PQ851486 |
| D0476 | 28.5 | DENV2 | 2II_F.1.1.2 | 95.93 | 2024-05-28 | Cali | PQ851487 |
| D0478 | 24.2 | DENV2 | 2II_F.1.1.2 | 100.0 | 2024-05-28 | Cali | PQ851488 |
| D0585 | 33.19 | DENV2 | 2II_F.1.1.2 | 65.44 | 2024-08-14 | Cali | * |
| D0586 | 33.3 | DENV2 | 2II_F.1.1.2 | 86.41 | 2024-08-15 | Cali | PQ851489 |
| D0587 | 31.47 | DENV2 | 2II_F.1.1.2 | 89.35 | 2024-08-08 | Cali | PQ851490 |

medRxiv preprint doi: https://doi.org/10.1101/2025.08.07.25333238; this version posted August 26, 2025. The copyright holder for this preprint (which was not certified by peer review) is the author/funder, who has granted medRxiv a license to display the preprint in perpetuity.
It is made available under a CC-BY 4.0 International license .

**Notes:**

* %Coverage below the threshold for submission; † Suspected co-infection or cross-contamination
